# Supplementary material for: Identification of single nucleotide polymorphisms (SNPs) potentially associated with residual feed intake in Qinchuan beef cattle by hypothalamus and duodenum RNA-Seq data
Source: PeerJ. 2025 Apr 15;13:e19270. doi: 10.7717/peerj.19270 (PMC12007499; doi:10.7717/peerj.19270)
Supplement: Supplemental Information 1 [file peerj-13-19270-s001.docx]

**Table S1.** Sample sequencing data quality control.

| Sample | Raw Reads | Clean Reads | Q30 | GC content | Sample | Raw Reads | Clean Reads | Q30 | GC content |
| --- | --- | --- | --- | --- | --- | --- | --- | --- | --- |
| Q_H1 | 106595686 | 106267846 | 98.36 | 52.5 | S_H1 | 29829057 | 29593065 | 96.33 | 48.5 |
| Q_H2 | 96280211 | 95978690 | 98.38 | 48.5 | S_H2 | 28291488 | 28078920 | 96.51 | 49.5 |
| Q_H3 | 102752595 | 102406632 | 98.21 | 50.0 | S_H3 | 26454721 | 26236700 | 96.23 | 50.0 |
| Q_H4 | 96884491 | 96577622 | 98.38 | 51.5 | S_H4 | 27856701 | 27621250 | 96.25 | 49.0 |
| Q_H5 | 90337801 | 90057236 | 98.18 | 49.0 | S_H5 | 26507574 | 26289725 | 96.30 | 49.0 |
| Q_L1 | 97164931 | 96840251 | 98.29 | 53.5 | S_L1 | 26298753 | 26083282 | 96.32 | 48.5 |
| Q_L2 | 97655285 | 97192880 | 97.49 | 52.0 | S_L2 | 26095774 | 25859356 | 96.38 | 49.0 |
| Q_L3 | 107574531 | 107194301 | 98.36 | 53.5 | S_L3 | 27804433 | 27575444 | 96.23 | 49.5 |
| Q_L4 | 119971329 | 119583908 | 98.28 | 54.0 | S_L4 | 28935372 | 28688038 | 96.31 | 49.0 |
| Q_L5 | 107574531 | 107194301 | 98.36 | 53.5 | S_L5 | 28702816 | 28481528 | 96.38 | 49.5 |
