# Supplementary material for: Identification of single nucleotide polymorphisms (SNPs) potentially associated with residual feed intake in Qinchuan beef cattle by hypothalamus and duodenum RNA-Seq data
Source: PeerJ. 2025 Apr 15;13:e19270. doi: 10.7717/peerj.19270 (PMC12007499; doi:10.7717/peerj.19270)
Supplement: Supplemental Information 2 [file peerj-13-19270-s002.docx]

Table S2: Genomic comparison statistics of sequenced reads.

| Sample | Uniquely Mapped Reads | Multiple Mapped Reads | Sample | Uniquely Mapped Reads | Multiple Mapped Reads |
| --- | --- | --- | --- | --- | --- |
| Q_H1 | 88.01% | 8.17% | S_H1 | 93.81% | 3.18% |
| Q_H2 | 90.76% | 5.84% | S_H2 | 93.15% | 3.50% |
| Q_H3 | 87.49% | 8.51% | S_H3 | 92.73% | 3.89% |
| Q_H4 | 87.53% | 8.59% | S_H4 | 93.23% | 3.31% |
| Q_H5 | 91.38% | 4.54% | S_H5 | 93.79% | 3.13% |
| Q_L1 | 81.07% | 14.30% | S_L1 | 93.33% | 3.51% |
| Q_L2 | 89.60% | 6.70% | S_L2 | 92.88% | 3.42% |
| Q_L3 | 87.00% | 8.81% | S_L3 | 92.52% | 4.01% |
| Q_L4 | 85.19% | 5.88% | S_L4 | 91.66% | 4.39% |
| Q_L5 | 87.00% | 8.81% | S_L5 | 92.50% | 3.88% |
